# Novel Potentials of the DPP-4 Inhibitor Sitagliptin against Ischemia-Reperfusion (I/R) Injury in Rat Ex-Vivo Heart Model

**DOI:** 10.3390/ijms19103226

**Published:** 2018-10-18

**Authors:** Amin Al-awar, Nikoletta Almási, Renáta Szabó, Istvan Takacs, Zsolt Murlasits, Gergő Szűcs, Szilvia Török, Anikó Pósa, Csaba Varga, Krisztina Kupai

**Affiliations:** 1 Department of Physiology, Anatomy and Neuroscience, Faculty of Science and Informatics, Szeged University, H-6726 Szeged, Hungary; amin@expbio.bio.u-szeged.hu (A.A.); almasi@expbio.bio.u-szeged.hu (N.A.); szaborenata88@gmail.com (R.S.); taki.biotech@gmail.com (I.T.); gergoszucs84@gmail.com (G.S.); tszilvia@bio.u-szeged.hu (S.T.); paniko@bio.u-szeged.hu (A.P.); vacs@bio.u-szeged.hu (C.V.); 2 Laboratory Animal Research Center, College of Arts and Sciences, Qatar University, Doha 2713, Qatar; zmurlasits@qu.edu.qa; 3 Department of Physiology, Anatomy and Neuroscience, Interdisciplinary Excellence Center, Szeged University, H-6726 Szeged, Hungary

**Keywords:** ischemia-reperfusion injury, infarct size, dipeptidyl-peptidase-4, DPP-4 inhibitors, NOS activity, transient receptor potential channels, Calcitonin gene related peptide, endothelial nitric oxide synthase

## Abstract

Dipeptidyl peptidase-4 (DPP-4) inhibitors are a class of oral anti-diabetic drugs, implicated in pleiotropic secondary cardioprotective effects. The aim of the study was to unveil the unknown and possible cardioprotective targets that can be exerted by sitagliptin (Sitg) against ischemia-reperfusion (I/R) injury. Male wistar rats received 2 weeks’ Sitg oral treatment of different doses (25, 50, 100, and 150 mg/kg/day), or saline as a Control. Hearts were then isolated and subjected to two different I/R injury protocols: 10 min perfusion, 45 min regional ischemia, and 120 min reperfusion for infarct size (IS) measurement, or: 10 min perfusion, 45 min regional ischemia and 10 min reperfusion for biochemical analysis: nitric oxide synthases (NOSs) and DPP-4 activity, glucagon-like peptide-1 (GLP-1), Calcium, transient receptor potential vanilloid (TRPV)-1 and calcitonin gene-related peptide (CGRP) levels, transient receptor potential canonical (TRPC)-1 and e-NOS protein expression. NOS inhibitor (l-NAME) and TRPV-1 inhibitor (Capsazepine) were utilized to confirm the implication of both signaling mechanisms in DPP-4 inhibition-induced at the level of IS. Findings show that Sitg (50 mg) resulted in significant decrease in IS and DPP-4 activity, and significant increase in GLP-1, NOS activity, e-NOS expression, TRPV-1 level and TRPC-1 expression, compared to controls. Results of CGRP are in line with TRPV-1, as a downstream regulatory effect. NOS system and transient receptor potential (TRP) channels can contribute to DPP-4 inhibition-mediated cardioprotection against I/R injury using Sitagliptin.

## 1. Introduction

Cardiovascular disorders, mainly ischemic heart disease, are the major cause of morbidity and mortality in patients with diabetes mellitus [1]. Reestablishing blood flow is considered to be the best therapeutic strategy and conventional remedy in myocardial infarction to prevent the myocardium from further damage, but nevertheless apoptosis of cardiomyocytes is inevitable in ischemia/reperfusion (I/R) injury [2]. The increasing prevalence of myocardial infarction means that researchers and pharmacologists have high interest in investigating new therapeutic agents that can reduce myocardial infarction (MI) and cardiac damage by I/R injury [3].

Oral dipeptidyl peptidase-4 (DPP-4) inhibitors have recently been introduced as anti-hyperglycemic drugs [4] that can exert either beneficial [5] or harmful [6] pleiotropic effects on the cardiovascular system. DPP-IV inhibitors prevent the degradation of the incretin hormones, namely glucagon-like peptide-1 (GLP-1) [7] that regulates glucose homeostasis after food ingestion [8]. Activation of GLP-1 by DPP-4 inhibitors and GLP-1 analogs, limits myocardial infarct size (IS) and causes upregulation in intracellular cascades like protein kinases, Akt/P-Akt, and extracellular signal-regulated kinases (ERK1/2) with protective profile against ischemia-reperfusion injury [4]. However, mechanisms of protection are still unclear, and a protective drug against infarct size is still unknown.

Nitric oxide synthases (NOSs) are enzymes catalyzing the production of nitric oxide (NO) from l-arginine, identified with 3 distinct isoforms (n-NOS, e-NOS and i-NOS) [9]. Endothelial NOS (e-NOS) have been identified with high abundancy in cardiomyocytes, and to be downregulated during prolonged myocardial ischemia [10,11]. Few studies addressed the mediated cardioprotective effect of DPP-4 inhibitors by NOS upregulation, however it is unknown whether sitagliptin exerts the same protective effect.

**Figure 1:**
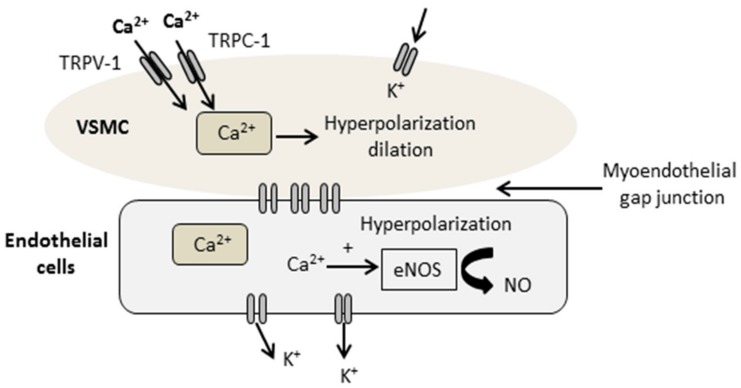
Schematic diagram showing the activation of the non-selective cation channels, transient receptor potential canonical (TRPC-1), increasing calcium (Ca^2+^) influx into the vascular smooth muscle cells (VSMC) and endothelial cells. Displayed data suggests that calcium influx induces the upregulation of endothelial nitric oxide synthase (e-NOS) and nitric oxide (NO) release, promoting endothelial protective effects.

Transient receptor potential (TRP) channels gained vast attention as a superfamily of non-selective and non-voltage gated ion channels functioning as cell signaling/sensory transducers [12]. Among the six subfamilies of TRP (transient receptor potential ankyrin (TRPA), transient receptor potential canonical (TRPC), transient receptor potential vanilloid (TRPV), transient receptor potential melastatin (TRPM), transient receptor potential polycystic (TRPP), and transient receptor potential mucolipidosis (TRPML)), the canonical and vanilloid TRP (TRPC and TRPV) channels are the most commonly localized and essential Ca^2+^-permeable channels in vascular endothelial cells, aorta, atria, ventricles, coronary blood vessels and sensory nerves innervating the heart [13,14,15], regulating Ca^2+^ through its direct effect as entry channels in plasma membrane, or by changing membrane potentials towards a significant driving force for Ca^2+^ entry [13,16] (Figure 1). These channels play a fundamental role in mediating ischemia-reperfusion injury and regulating cardioprotective signaling, in addition to the reduction in TRPC degradation and over-expression that were shown to have a protective effect against brain ischemia-reperfusion injury [17,18]. Calcitonin gene-related peptide (CGRP), is a major transmitter in capsaicin sensitive sensory nerves, and widely distributed in cardiac tissues [19]. The latter was extensively studied and its upstream regulator TRPV-1, with little evidence addressing its cardioprotective function [19,20], and no any data addressing its regulatory effect using DPP-4 inhibitors.

Therefore, the aim of our study was to investigate the cardioprotective effect of sitagliptin, a DPP-4 inhibitor, through its limiting effect on ischemic cardiomyocytes, and explore the underlying mechanisms in *ex-vivo* working heart model. We hypothesized that NOS and TRP channels can be potent mediators in this cardioprotection, and specific inhibitors were used for this purpose. To test our hypothesis, rats were pre-treated orally with sitagliptin for 2 weeks before inducing myocardial I/R.

## 2. Results

### 2.1. DPP-4i Decreased the Infarct Size in Heart Tissues of Sitg (50 mg) Group

After two weeks of daily oral administration of four different doses of the same DPP-4 inhibitor (Sitagliptin (Sitg)), prior to ischemia-reperfusion injury and after subjecting the heart tissues to 45 min of regional ischemia and 120 min of reperfusion, the Sitg (50 mg/kg/day) treated group exhibited a significant decrease in infarct size (22.20 ± 2.03%) compared to the Control group (44.89 ± 4.02%). This cardioprotective effect was dose-dependent, and the effective dose was used in further experiments and measurements.

**Figure 2:**
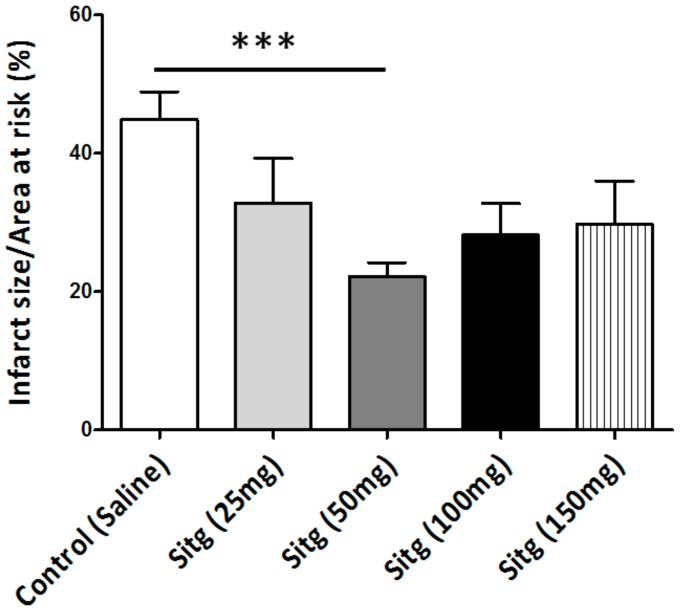
Effect of different doses of Sitagliptin (Sitg) on infarct size (expressed in %). Results are shown as (Mean ± Standard error of mean (SEM)); (*n* = 8–16 animals/group). Statistical significance: *** *p* < 0.001 relative to the Control group. Sitg (50 mg) exhibited a cardioprotective effect against ischemia-reperfusion (I/R) injury, while no significance was reported in other doses (25, 100, and 150 mg/kg/day).

The area of infarction is expressed as the percentage of infarct size over the area at risk (Figure 2).

### 2.2. DPP-4i Normalized DPP-4 Activity and Enhanced GLP-1 Level

**Figure 3:**
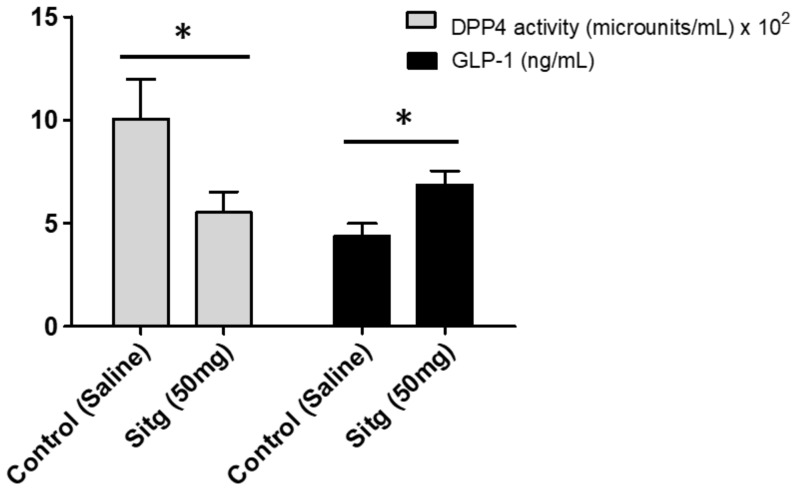
Changes in Dipeptidyl peptidase-4 (DPP-4) enzyme activity (expressed in microunits/mL) and Glucagon-like peptide 1 (GLP-1; expressed in ng/mL) in the heart tissues of Sitagliptin (Sitg 50 mg) treated animal groups. Data are represented as (Mean ± SEM); (*n* = 4–10 animals/group). Statistical significance: * *p* < 0.05 compared to the Control group.

Sitagliptin reduced DPP-4 activity (552.32 ± 100.02 microunits/mL) by 50% in the Sitg (50 mg/kg) treated group, compared to the Control group (1005.92 ± 190.96 microunits/mL). Measurements of GLP-1 level from heart tissues subjected to brief reperfusion (10 min), revealed a significant increase (44.98 ± 4.02 ng/mL) in the Sitg (50 mg) treated group compared to the Controls (22.20 ± 2.03 ng/mL). Results are shown in Figure 3.

### 2.3. DPP-4i Increased TRPV-1 and CGRP Levels in Heart Tissues of Sitg (50 mg)

**Figure 4:**
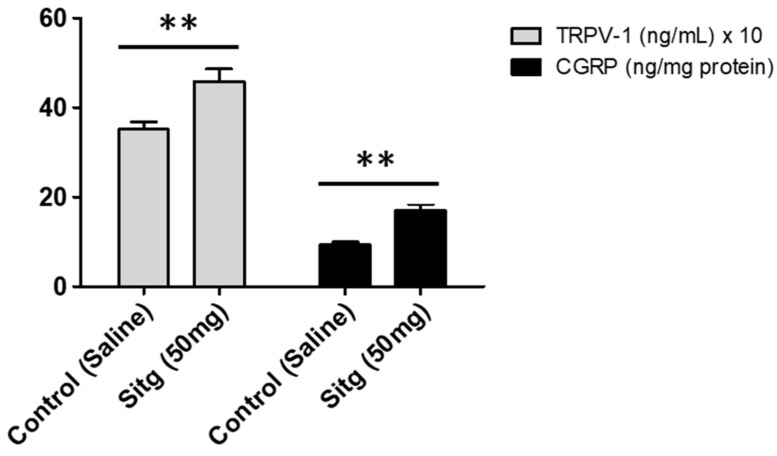
Effect of Sitagliptin treatment on TRPV-1 (expressed in ng/mL) and CGRP (ng/mg protein) ischemic cardiac tissue levels, compared to the Control animal group. A clear significant increase is observed in both proteins levels observed comparing the treated group to the Control (** *p* < 0.01). Data are illustrated as (Mean ± SEM); (*n* = 5–10 animals/group).

Convincingly, a significant increase in TRPV-1 level (458.49 ± 27.62 ng/mL) was observed in the Sitg (50 mg) group compared to the Control group (351.04 ± 17.40 ng/mL). Results of CGRP measurements are in line with that of TRPV-1, showing a clear increase in CGRP level (16.91 ± 1.57 ng/mg protein) vs. (9.36 ± 0.65 ng/mg protein), in the Sitg (50 mg) and Control groups respectively. Results are displayed in Figure 4.

### 2.4. DPP-4i Augmented Cardiac Calcium (Ca^2+^) Content in Hearts of Sitg (50 mg) Group

**Figure 5:**
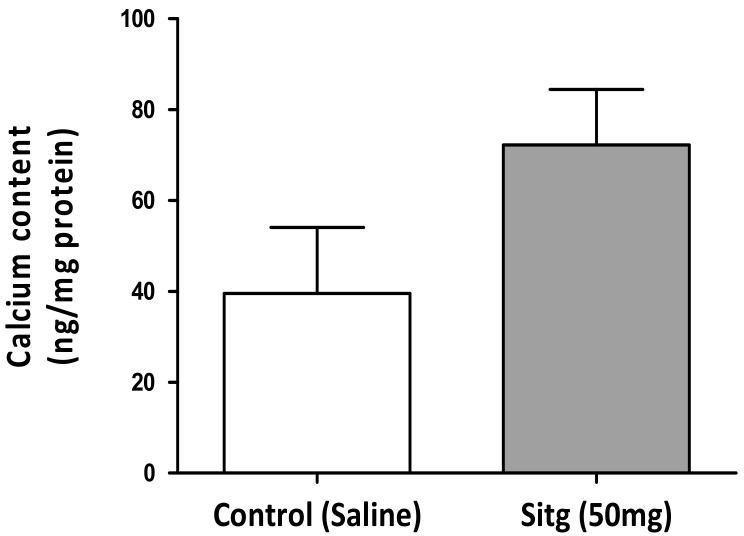
Changes in calcium content of cardiac tissues excised from the Sitagliptin (Sitg (50 mg); *n* = 7) treated animals and the Control ones (*n* = 4). The bar chart displays an increase in calcium concentration in Sitg (50 mg) group, and values presented are in terms of (Mean ± SEM).

To determine whether the ischemic cardiac calcium concentration was affected by the DPP-4 inhibitor (Sitagliptin) treatment, a colorimetric calcium detection assay kit was used, and obtained findings indicated an increase in calcium content in heart tissues assigned to drug therapy (72.23 ± 12.19 ng/mg protein) vs. Controls (39.55 ± 14.49 ng/mg protein) (Figure 5).

### 2.5. DPP-4i Positively Affected TRPC-1 Protein Expression

**Figure 6:**
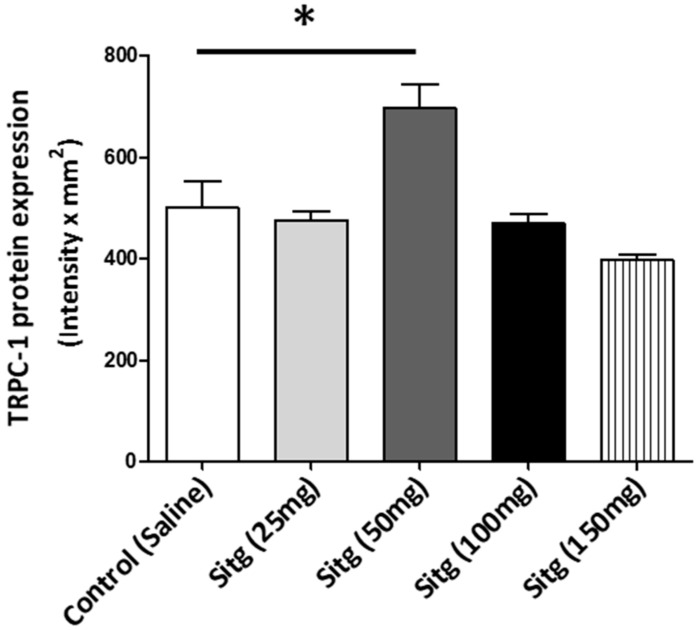
Upregulation of TRPC-1 protein expression level (expressed in Intensity × mm^2^) in the heart tissues of Sitagliptin (Sitg (50 mg); *n* = 5) treated group vs. Control (*n* = 6). Data are in terms of (Mean ± SEM). Statistical significance: * *p* < 0.05.

The difference in TRPC-1 protein expression level between the different treated groups with different doses of sitagliptin is presented in Figure 6. Sitg (50 mg) treated group showed a higher level of TRPC-1 expression (697.80 ± 46.37 Intensity × mm^2^) in comparison with the Control group (499.93 ± 52.28 Intensity × mm^2^), while no significant difference was observed in other doses.

### 2.6. DPP-4i Upregulated e-NOS Protein Expression and cNOS Activity in Heart Tissues of Sitg (50 mg)

#### 2.6.1. e-NOS Protein Expression

**Figure 7:**
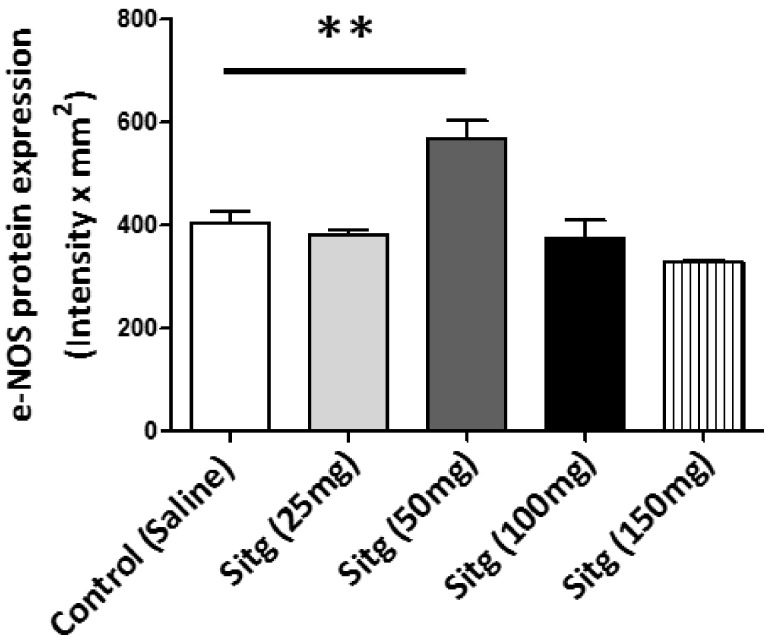
Increase in endothelial nitric oxide synthase (e-NOS (*n* = 5–6)) protein expression in ischemic cardiac tissues from Sitagliptin (Sitg (50 mg)) treated group compared to Control (** *p* < 0.01). Values are expressed in (Intensity × mm^2^). Presented data are (Mean ± SEM).

Protein expression of endothelial NOS (e-NOS) isoform, which is also known as nitric oxide synthase-3 (NOS-3), as determined by Western blot in different treated groups, is shown in Figure 7. Obviously, e-NOS expression was significantly increased in the Sitg (50 mg) treated animals (568.95 ± 34.74 Intensity × mm^2^), in comparison with the Control ones (403.19 ± 24.86 Intensity × mm^2^); however, comparing other doses to the controls showed no significant change.

##### 2.6.2. cNOS Activity

**Figure 8:**
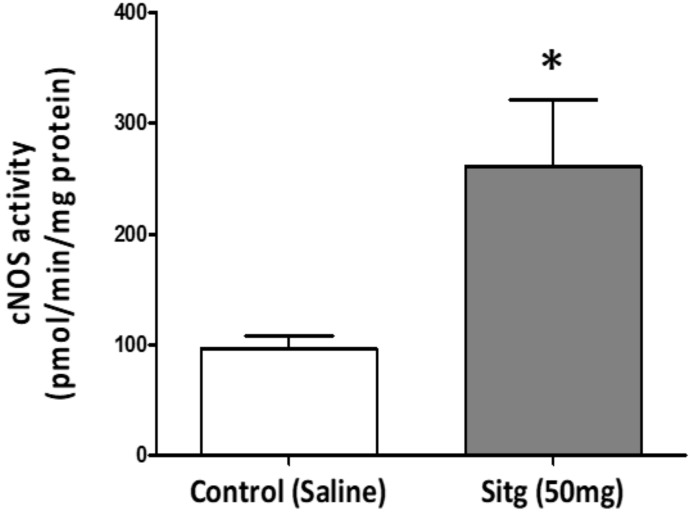
Increase in nitric oxide synthase activity (cNOS (*n* = 8)) in ischemic cardiac tissues from Sitagliptin (Sitg (50 mg)) treated group compared to Control (* *p* < 0.05). Values are expressed in (pmol/min/mg protein). Presented data are (Mean ± SEM).

Two weeks of daily oral treatment with Sitagliptin (50 mg), followed by brief reperfusion (45 min occlusion and 10 min reperfusion) of the coronary artery showed a significant increase in cNOS activity (260.87 ± 60.86 pmol/min/mg protein) relative to the Control group (96.47 ± 11.71 pmol/min/mg protein) (Figure 8).

### 2.7. l-NAME Inhibited NOS-Mediated Cardioprotection against Infarct

**Figure 9:**
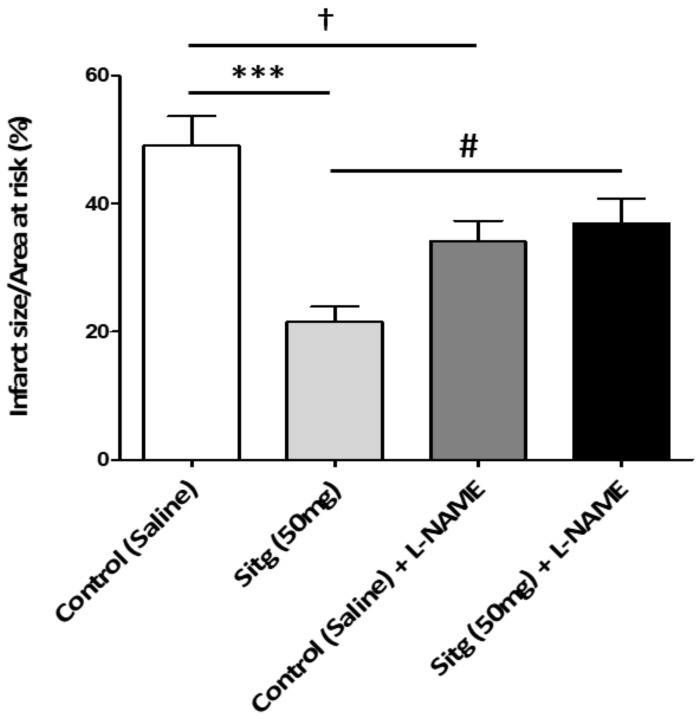
Loss of cardioprotective effect mediated by NOS and increase in infarct size with intraperitoneal injection of NOS-inhibitor (l-NAME), infarct size expressed in (%). Comparing Control (Saline, *n* = 12) and Sitagliptin (Sitg (50 mg), *n* = 10) groups shows a significant decrease in infarct size (*** *p* < 0.001), while this protective effect was abolished comparing the Sitg (50 mg) + l-NAME (Sitagliptin 50 mg + Nω-Nitro-l-arginine methyl ester hydrochloride (i.p), *n* = 11) group with the Sitg (50 mg, *n* = 10) treated group (# *p* < 0.05), which means that cardioprotective effect of Sitagliptin against infarction is mediated through NOS. A significant decrease († *p* < 0.05) in infarct size was also observed in Control (Saline) + l-NAME (Saline + Nω-Nitro-l-arginine methyl ester hydrochloride (i.p), *n* = 11) group, compared to Control (Saline). Data plotted as (Mean ± SEM).

To evaluate the cardioprotective mechanism of the DPP-4i (Sitagliptin)—mediated by nitric oxide synthase (NOS), rats were treated with l-NAME (NOS-inhibitor) and the size of infarction was assessed. Myocardial infarct size quantifications as a percentage of the left ventricle (LV) and the area at risk are shown in Figure 9. Results from the Sitg (50 mg) treated group matches the previous ones, showing 3-folds significant reduction (21.56 ± 2.41%), compared to the Controls (49.09 ± 4.60%). However, this protective effect disappeared in the Sitg (50 mg) + l-NAME—treated animal group (36.99 ± 3.82%) vs. the animal group treated with Sitg (50 mg) alone (21.56 ± 2.41%). l-NAME also decreased infarct size (21.56 ± 2.41%) in Control (Saline) + l-NAME group, compared to Control (Saline) (49.09 ± 4.60%).

### 2.8. Capsazepine Inhibited TRPV-1-Mediated Cardioprotection against Infarct

**Figure 10:**
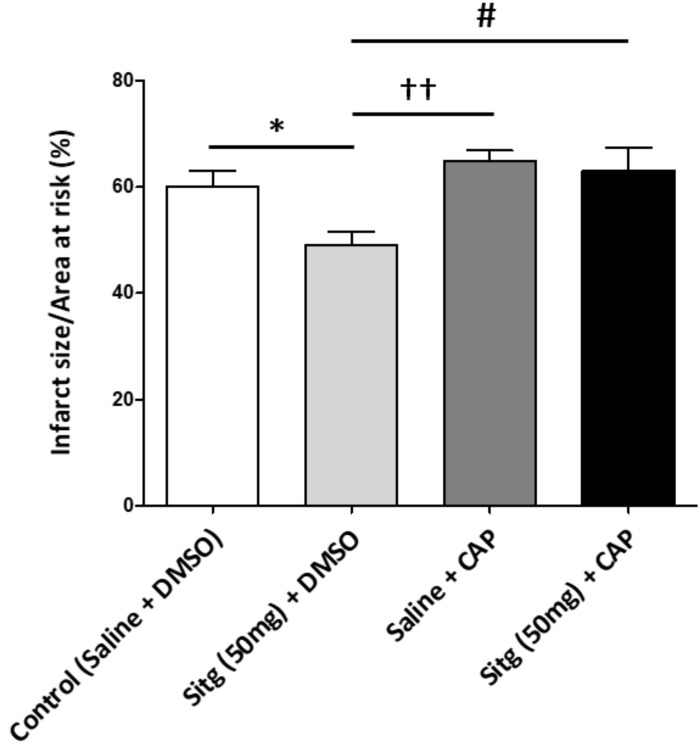
Loss of cardioprotective effect mediated by TRPV-1 and increase in infarct size with intraperitoneal injection of TRPV-1-inhibitor (Capsazepine), Infarct size expressed in (%). Comparing the 2 groups, Control (Saline) + DMSO (Control (Saline) + Dimethyl sulfoxide (i.p), *n* = 7) and Sitg (50 mg) + DMSO (Sitagliptin 50 mg + Dimethyl sulfoxide (i.p), *n* = 6), shows a significant decrease in infarct size (* *p* < 0.05), while this protective effect was abolished comparing the Sitg (50 mg) + CAP (Sitagliptin 50 mg + Capsazepine (i.p), *n* = 8) group with the Sitg (50 mg) + DMSO treated group (# *p* < 0.05), which means that cardioprotective effect of Sitagliptin against infarction is mediated by TRPV-1. A significant difference (†† *p* < 0.01) was observed in Sitg (50 mg) + DMSO (Sitagliptin (50 mg) + Dimethyl sulfoxide (i.p), *n* = 6) group, compared to Saline + CAP (Saline + Capsazepine (i.p), *n* = 8). Data plotted as (Mean ± SEM).

The area of infarction was measured in the presence and absence of TRPV-1 inhibitor (Capsazepine), to test whether TRPV-1 is directly implicated in DDP-4i (Sitg)-mediated cardioprotection. Quantification of infarct size as a percentage of the left ventricle (LV) and the area at risk are shown in Figure 10. Animal group treated with Sitg (50 mg) + DMSO exhibited a clear decrease in infarct size (49 ± 2.50%) vs. the Control dimethyl sulfoxide (DMSO) group (62.38 ± 1.99%). However, TRPV-1 inhibition with Capsazepine blocked this cardioprotection in Sitg (50 mg) + CAP-treated group (63.01 ± 4.32%) compared to the group treated with Sitg (50 mg) + DMSO (49 ± 2.50%). A significant difference in infarct size was also observed in Saline + CAP group (64.81 ± 1.98%), compared to Sitg (50 mg) + DMSO (49 ± 2.50%).

## 3. Discussion

When the circulation is abruptly restored after a prolonged myocardial ischemia, this can lead to cardiomyocyte damage which is commonly referred to myocardial I/R injury, triggered by neutrophil accumulation, causing ROS production and cellular damage [21]. In the present study, 45 min of regional ischemia and 120 min of reperfusion in sustained I/R injury, revealed a significant percentage of infarction (infarct size), using triphenyltetrazolium chloride (TTC) staining. Accordingly, developing cytoprotective pharmacological strategies in the frame of limiting myocardial infarction by maintaining a proper blood flow to the ischemic myocardial region is one of the main focuses of preclinical and clinical research [22].

**Figure 11:**
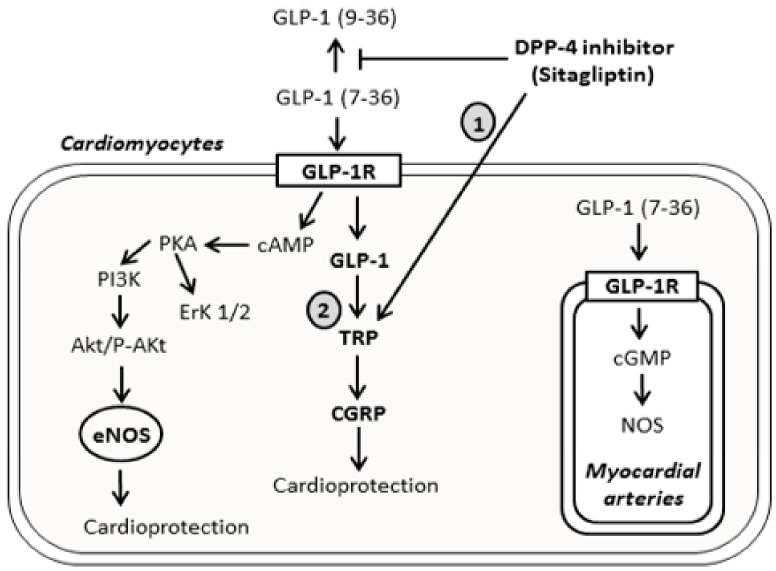
Schematic diagram showing the signaling pathways activated by the DPP-4 inhibitor sitagliptin, directly or upon binding to GLP-1 receptors. The diagram represents the traditional signaling mechanisms involved in cardioprotection, including cAMP/PKA, PI3K, Akt/P-Akt, ErK1/2, and cGMP, mediated NOS upregulation and e-NOS production. It also clarifies the novelty of this study (Upregulation of TRP channels and CGRP mediated by sitagliptin and GLP-1). Activation of TRP channels is either through the direct effect of DPP-4 inhibitor **(1)**, or through GLP-1R and GLP-1 activation (2). The arrow sign (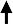) indicates mechanism’s activation, and the T bar sign (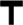) indicates the inhibitory effect.

Clinical and experimental animal investigations suggested that incretins, namely GLP-1, can exhibit pleiotropic cardioprotective potentials following myocardial ischemia (MI), via preserving the cardiomyocytes viability, increasing metabolic efficiency, and inhibiting the structural and functional cardiac remodeling [23]. DPP-4 is abundantly expressed in the cardiovascular system and endothelial cells [24], and blocking its activity by DPP-4 inhibitors can have advantageous cardiovascular outcomes, through upregulation of GLP-1 levels, inhibition of substrates of cardiovascular homeostasis, in addition to its impact on glucose metabolism [25]. According to Chinda et al. [26], acute administration of the DPP-4 inhibitor vildagliptin reduced infarct size by 44%. According to previous results, oral and intraperitoneal administration of sitagliptin at high doses, exerted a limiting effect on infarct size and triggered pro-survival signaling cascades (PI3K-Akt and ERK1/2) by GLP-1 upregulation, as responses to ischemia reperfusion (IR) injury [27]. Our study introduces the infarct size limiting effect of sitagliptin pretreatment at a dose of 50 mg, and induction of new biochemical markers (TRP/CGRP) preceded by GLP-1 activation (Figure 11).

The deleterious consequences of I/R injury can be a major cause of endothelial dysfunction, causing a reduction in endothelial nitric oxide synthase (e-NOS) expression, and maintaining adequate levels of e-NOS is cytoprotective [28,29]. Increased phosphorylation and activation of e-NOS was addressed using the DPP-4 inhibitor alogliptin [30]. Prolonged myocardial ischemia decreased NOS activity and e-NOS (NOS-3) protein expression [11], but the new findings of our results show an increase in e-NOS expression in ischemic hearts pretreated with 50 mg dose of Sitagliptin. l-NAME is a non-selective NOS inhibitor and inhibits the activities of the three NOS isoforms: e-NOS, inducible NOS (i-NOS) and neuronal NOS (n-NOS) [31]. In our experiments, the cardioprotective action of sitagliptin mediated by NOS was confirmed at the level of infarct size, using l-NAME. The decrease in infarct size in the group treated with Sitg (50 mg) was abrogated when compared to the group treated with sitagliptin and l-NAME (Sitg (50 mg) + l-NAME). Interestingly, l-NAME decreased infarct size when treated alone with saline (Saline + l-NAME), compared to the control (Saline) group, and this can be related to the effect of NOS inhibitor (l-NAME) in protecting rat hearts from I/R injury by decreasing OONO^−^ generation [32].

Studies on transient receptor potential channels revealed that the upregulation of TRPC and TRPV subfamilies contributes to the pathophysiology of vascular and cardiac tissues [33], with direct implication of increased TRPC-1 levels in cardiac hypertrophy [34]. Our results are not in agreement with the latter findings, showing a significant increase in TRPV-1 level and TRPC-1 expression post-ischemia-reperfusion injury, in cardiac tissues pretreated with 50 mg dose of sitagliptin. Administration of TRP inhibitor (Capsazepine), blocked the infarct size limiting effect of sitagliptin, showing that this protection is mediated by these channels. Stimulation of TRPV-1 promotes the release of CGRP, with accumulating data reporting the advantageous role of CGRP in enhanced myocardial contractility and increased heart rate [20], and this protective effect of TRPV-1 and CGRP is in concordance with measurements from our study. Without forgetting the activation of this cascade (TRPV-1/CGRP) during cardioprotective brief episodes (Pre-conditioning and Post-conditioning), against myocardial infarction in rat hearts [35,36], to the best of our knowledge, this study is the first to show the upregulation of the underlying pathway (TRPV-1/CGRP) using sitagliptin as a new targeting therapy in prolonged ischemia-reperfusion injury.

### Limitations

In the present study, the 50 mg dose of sitagliptin have shown a promising protective effect on myocardial IS and cardiomyocyte survival after ischemia-reperfusion injury *ex vivo*, which can be clinically relevant. Based on this encouraging result, we stretched our experiments to study the mechanisms and biochemical markers underlying this dose. In the clinical setting, the importance lies in the dose-protective effect of any drug; therefore, our focus was on the 50 mg dose only. However, it can be possible that high doses of sitagliptin reversed this protective effect, and investigating the underlying mechanisms in high doses can have clinical benefits by avoiding the use of detrimental doses. 

We studied the effect of sitagliptin in *ex vivo* I/R injury model, and have shown relevant resulting cardioprotective effects against myocardial infarction, by lowering infarct size and activation of new survival targeting pathway. However, using an *in vivo* I/R injury model with the same doses we have used in this study, can show additive protective effects and clinical relevance of sitagliptin.

Similar to our previous studies, we used 10 min brief reperfusion in our experiments for the purpose of biochemical measurements. Short reperfusion protocols preceded by coronary occlusions are commonly used to test the effect of drugs on ischemia-reperfusion injury in *ex vivo* rodent heart. It could be possible that measuring protein markers before reperfusion can have an unprecedented accuracy for the underlying molecular mechanisms that can predispose the heart tissue to resist reperfusion injury.

## 4. Materials and Methods

### 4.1. Drug Preparations

Sitagliptin filmtablets (Januvia 100 mg, Merk Sharp & Dohme Ltd., Hertfordshire EN11 9BU, Hertford Road, Hoddesdon, UK) were purchased and freshly dissolved in saline (0.9%) on a daily basis and before each treatment. The anesthetic agent Thiopental (Tiobarbital Braun, 0.5 g, B. Braun Medical SA, Barcelona, Carretera de Terrassa, Spain) was also dissolved in saline (0.9%). NOS-inhibitor (Nω-Nitro-l-arginine methyl ester hydrochloride (l-NAME)), purchased from Sigma Aldrich (St. Louis, MO, USA) and dissolved in physiological saline (0.9%), while TRPV-1 inhibitor (Capsazepine) was also purchased from Sigma Aldrich and dissolved in dimethyl sulfoxide (DMSO).

### 4.2. Animals and Experimental Design

This study conforms with the standards of the European Community guidelines for the Care and Use of Laboratory Animals, and all procedures were performed according to the protocols approved by the Institutional Ethical Animal Care and Use Committee of Szeged University, with the project identification code and date of approval (XX./4801/2015, 15 December 2015). Six to eight-week-old male wistar rats (body weight 200–300 g; Toxi-Coop Ltd., Dunakeszi, Hungary) were obtained and acclimatized for one week before the commencement of treatments. All animals were housed in our temperature-controlled animal facility (23 °C), maintained with a 12:12-h light–dark cycle with food and water provided ad libitum. Finally, our research work complies with the commonly-accepted “3Rs”: Replacement of animals by alternatives wherever possible, Reduction in number of animals used, and Refinement of experimental conditions and procedures to minimize the harm to animals.

**Figure 12:**
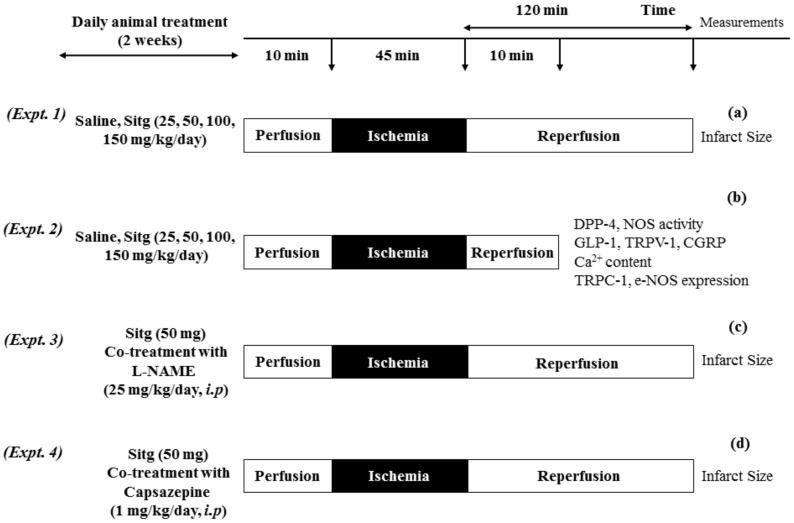
Diagram illustrating 4 different I/R experimental protocols. (**a**) Heart tissues subjected to 45 min ischemia and 120 min of reperfusion, after 2 weeks of oral animal treatment with Saline and different doses of Sitagliptin, for infarct size measurement. (**b**) Hearts subjected to 45 min ischemia and 10 min brief reperfusion, after which the animals received a 2 weeks’ oral administration of Saline and Sitg (50 mg), for further biochemical measurements. Infarct size measurement from heart tissues exposed to prolonged ischemia-reperfusion injury, after which the animals received a 2 weeks’ co-treatment of Saline, Sitg (50 mg) and intraperitoneal injection of NOS-inhibitor (l-NAME) (**c**). In the 4th experimental protocol (**d**), the inhibitory effect of TRPV-1 against ischemia-reperfusion (IR) injury was assessed by heart infarct size measurement at the end of a prolonged reperfusion-injury, and after co-treating the animals intraperitoneally with Capsazepine (TRPV-1 inhibitor).

Animals were assigned into 4 different experiments:

**Experiment 1.** To determine the most effective dose (kg^−1^day) of Sitagliptin (Sitg), animals were randomly divided into 5 groups: (Control (Saline), Sitg (25 mg), Sitg (50 mg), Sitg (100 mg) and Sitg (150 mg), *n* = 8–16). The daily oral administration of different drug doses or its vehicle (Saline) lasted for two weeks. At the end of the treatment, the whole-heart preparation and ischemia-reperfusion (I/R) injury protocol started. Animals were anesthetized with thiopental (i.p. 100 mg/kg), heart tissues were excised and immediately placed in ice-cold saline (0.9%), mounted and ligated through the aorta into the cannula (ex vivo) of a modified Langendorff Apparatus System, and perfused with 37 °C Krebs buffer (118 mM NaCl, 4.70 mM KCl, 2.50 mM CaCl_2_, 1.18 mM MgSO_4_, 1.18 mM KH_2_PO_4_, 5.50 mM glucose and 25 mM NaHCO_3_ and gassed with 95% O_2_ and 5% CO). Hearts were exposed to 10 min perfusion, 45 min prolonged regional ischemia by left anterior descending (LAD) coronary artery occlusion, followed by 120 min reperfusion. At the end of the experiment, heart tissues were re-ligated from the left anterior descending (LAD) coronary artery, and the area at risk (AAR) was stained with Evans blue dye via the aortic root. Hearts were weighed and stored at −20 °C for further triphenyltetrazolium chloride (TTC) staining. The Sitg (50 mg) dose was used in our further experiments as the effective dose (Figure 12a).

**Figure 13:**
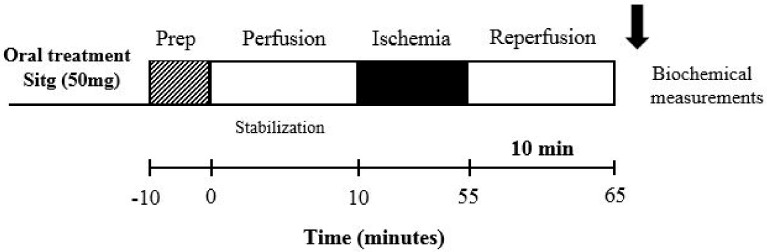
Schematic diagram clarifying the ischemia-reperfusion (I/R) protocol used with brief reperfusion time (10 min) for the purpose of biochemical measurements in experiment 2.

**Experiment 2.** For the purpose of in vitro laboratory measurements, another set of experiments was carried out by assigning only two animal groups (Control (Saline) and Sitg (50 mg/kg/day), *n* = 10), and the oral daily treatment lasted for two weeks. At the end of the treatment, the whole-heart preparation and ischemia-reperfusion (I/R) injury protocol were performed. Rats were randomly anesthetized with thiopenthal (i.p. 100 mg/kg), heart tissues were fastly excised, placed in ice-cold saline (0.9%), mounted and ligated through the aorta into the cannula (ex vivo) of a modified Langendorff Apparatus System, and perfused with the same Krebs buffer mentioned in Experiment 1. Hearts were exposed to 10 min perfusion, 45 min prolonged regional ischemia by occluding the left anterior descending (LAD) coronary artery, followed by a brief reperfusion for 10 min. At the end of the experiment, heart tissues were weighed, directly clamped and stored at −80 °C for further biochemical analyses (Figure 12b and Figure 13).

**Experiment 3.** To confirm the involvement of NOS in Sitg (50 mg)-mediated cardioprotection against ischemia-reperfusion (IR) injury, four different animal groups (Control (Saline), Sitg (50 mg), Control (Saline) + l-NAME, and Sitg (50 mg) + l-NAME, *n* = 10–12) were studied. The Control (Saline) and Sitg (50 mg) animal groups received the same daily oral treatment as in Experiment 1, while the other two groups were co-treated intraperitoneally (i.p) with a specific NOS inhibitor (l-NAME, 25 mg/kg/day) [37], three hours post-oral administration of Saline and Sitg (50 mg). At the end of the treatment, the same anesthesia, hearts excision, whole-heart preparation and ischemia-reperfusion (I/R) injury protocols (10 min perfusion, 45 min prolonged regional ischemia and 120 min reperfusion, ex vivo), coronary artery re-ligation, and cardiac tissue staining procedures mentioned in Experiment 1 were performed for all groups (Figure 12c).

**Experiment 4.** To evaluate the inhibitory effect of TRPV-1 on infarct size (IS), another four sets of animals (Control (Saline) + DMSO, Sitg (50 mg) + DMSO, Control (Saline) + CAP, and Sitg (50 mg) + CAP, *n* = 5–8) received the same daily oral treatments, and co-treated daily using intraperitoneal (i.p) injections with either DMSO or TRPV-1 inhibitor (Capsazepine (CAP), 1 mg/kg/day) [38], three hours post-oral treatments, for two weeks. At the end of the treatment, the same anesthesia, heart excision, whole-heart preparation and ischemia-reperfusion (I/R) injury protocols (10 min perfusion, 45 min prolonged regional ischemia and 120 min reperfusion, ex vivo), coronary artery re-ligation, and cardiac tissue-staining procedures mentioned in Experiment 1 were performed for all groups (Figure 12d).

### 4.3. Tissue Staining and Infarct Size Measurement

**Figure 14:**
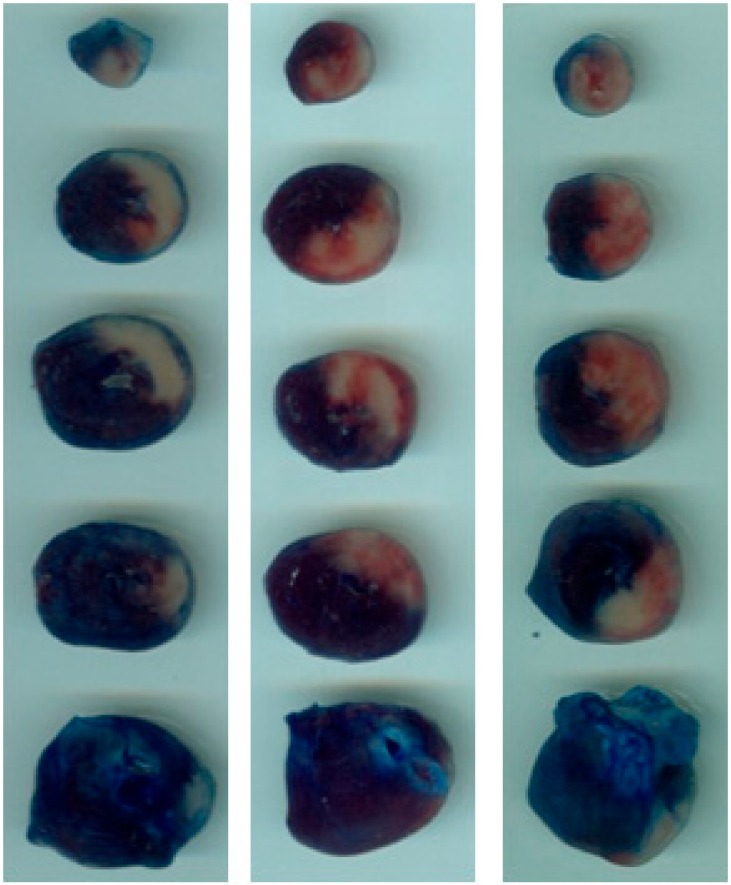
Representative photographs of transversely sectioned Evans-blue perfused, triphenyltetrazolium chloride (TTC)—stained heart tissues, outlining the area at risk (AAR; sum of white and red area); blue, healthy viable tissue; pale white, infarcted tissue. Myocardial infarct area (IS; white) was measured post-myocardial ischemia-reperfusion and TTC staining, in different treated groups and Controls.

At the end of each prolonged reperfusion phase (120-min), the left anterior descending (LAD) coronary artery was re-ligated, and the risk zone was stained with Evans blue dye via the aortic root. Hearts were frozen, transversely sectioned into (5–6 slices; 2-mm thickness) from the apex to the base, and incubated in 1% triphenyltetrazolium chloride (TTC) for 10 min at 37 °C. After incubation, tissue sections were fixed for 10 min in 10% formalin, and then placed for 30 min in phosphate buffer (pH 7.4). All sections were mounted on glass slides, images were captured with a digital camera, and an ImageJ 1.34 software was used to measure the infarcted areas. The scar was measured in each section by an investigator who was blinded to the identity of the sections (Figure 14).

### 4.4. DPP-4 Activity Test

The cardiac DPP4 (CD26) activity in Control and Sitg (50 mg) treated animal groups was assessed using DPP4 activity assay kit and according to the manufacturer’s guidelines (Sigma Aldrich, 3050 Spruce Street, St. Louis, MO 63103, USA). A 10 mg of heart tissues were homogenized in ice-cold DPP4 Assay Buffer, centrifuged at 13,000× *g*, at 4 °C for 10 min, and supernatants were harvested. Standard and sample fluorescence intensity (FLU) measurements (*λ*ex = 360/*λ*em = 460 nm) were carried after five min of incubation at (37 °C) in 96-well black plates specific for fluorescence assays, using a fluorescence multiwell plate reader (Fluorometer). Incubation and measuring cycles were repeated, until the most active sample was near to or greater than the value of the highest standard (100 pmole/well). Results are expressed as (microunit/mL).

### 4.5. Nitric Oxide Synthase (NOS) Activity

NOS activity was measured by quantifying the conversion of [^14^C]-labeled l-arginine to citrulline by a previously-described method with some minor modifications [39]. Heart tissues were homogenized with Ultra-Turrax T25 (13,500/s; twice for 30 s) in ice-cold 10 mM *N*-[2-hydroxyethyl] piperazine-*N*′-[2-ethanesulfonic acid] (HEPES, Sigma-Aldrich), 32 mM sucrose (Sigma-Aldrich), 1 mM dithiothreitol (DTT, Sigma-Aldrich), 0.1 mM EDTA, 10 μg/mL soybean trypsin inhibitor (Sigma-Aldrich), 10 μg/mL leupeptin (Sigma-Aldrich), and 2 μg/mL aprotinin (Sigma-Aldrich), at pH 7.4. Supernatants were collected by centrifugation for (30 min, 20,000× *g*, 4 °C). Samples (40 μL) were incubated for 10 min at 37 °C with 100 μL of assay buffer (50 mM KH_2_PO_4_, 1.0 mM MgCl2, 50 mM l-valine, 0.2 mM CaCl_2_, 1.0 mM dithiotreitol (DTT), 1.0 mM l-citrulline, 15.5 nM l-arginine, 30 μM flavin adenine dinucleotide, 30 μM flavin mononucleotide, 30 μM tetrahydro-l-biopterin dihydrochloride, 450 μM β-nicotinamide adenine dinucleotide phosphate (β-NADPH), and 12 pM [^14^C]-l-arginine monohydrochloride (all from Sigma-Aldrich, Budapest, Hungary). The reaction was terminated by addition of 0.5 mL of a 1:1 (*v*/*v*) suspension of ice-cold DOWEX (Dowex 50WX8 hydrogen form 100-200 mesh, Sigma Aldrich) in distilled water. The mixture was re-suspended by adding 850 μL of ice-cold distilled water, supernatant (970 μL) was removed and radioactivity was determined by scintillation counting. The Ca^2+^ dependence of the NOS activity was determined by addition of 10 μL of ethylene glycol-bis (β-aminoethyl ether) tetraacetic acid (EGTA; 1 mM, Sigma-Aldrich). NOS activity was confirmed by inhibition with 10 μL of Nω-nitro-l-arginine methyl ester (l-NNA; 3.7 mM, Sigma-Aldrich). The level of inducible NOS (i-NOS) was defined as the extent of citrulline formation that was inhibited by l-NNA, but not by EGTA. The constitutive NOS (cNOS) activity was calculated from the difference between the extent of citrulline formation inhibited by EGTA and the total activity. As the nature of the cNOS isoform (e-NOS or n-NOS) was not determined, this activity is referred to as cNOS. NOS activity is expressed as (pmol/min/mg protein).

### 4.6. ELISA Measurements (GLP-1, TRPV-1 and CGRP)

A double-antibody sandwich ELISA kits specific for rat GLP-1, TRPV-1 and CGRP measurements were purchased from the same company (SunRed Biotechnology, Shanghai, China), and with the same homogenization buffer (Phosphate Buffer Saline (PBS), pH 7.2–7.4) and homogenization procedure (Homogenization by Ultra Turrax T8, 20 min centrifugation at 2000–3000 rpm). The whole tissue sample preparation procedure was done on ice. The three parameters were measured according to the manufacturer’s instructions and protocols, and optical densities (OD) were determined at *λ* = 450 nm. Results were expressed in (ng/mL) for GLP-1 and TRPV-1, and (ng/L) for CGRP.

### 4.7. Calcium (Ca^2+^) Content Test

A Colorimetric Calcium Detection Assay Kit (Abcam, Cambridge, UK) was used to determine the calcium (Ca^2+^) concentration. Samples were homogenized on ice using PBS + 0.1% NP-40, centrifuged at a maximum speed for 2–5 min. Supernatants were collected, and measurements were performed according to the provided procedure. Optical densities (OD) were detected at (*λ* = 575 nm). Results are expressed in (ng/mg protein).

### 4.8. TRPC-1 and e-NOS Protein Expression by Western Blotting

**Figure 15:**
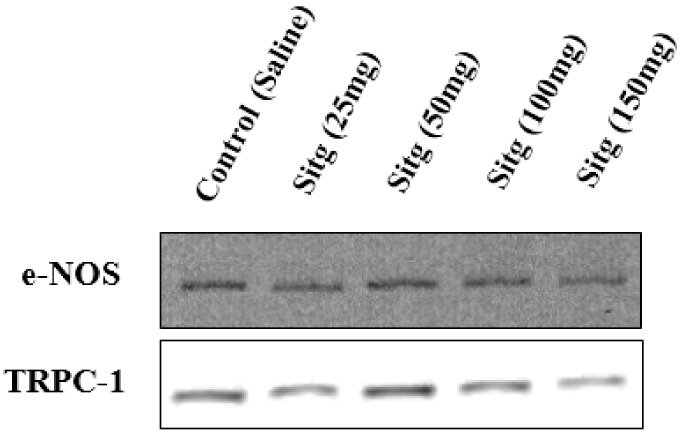
Expression of e-NOS and TRPC-1 proteins in ischemic heart tissues treated with different doses of sitagliptin (Sitg (25, 50, 100, & 150 mg)), compared to the control (Saline) group. The blots show that both proteins are significantly expressed in sitagliptin (50 mg) treated groups.

Measured heart tissues were homogenized by Ultra-Turrax T25 (13,500/s; twice for 30 s) with ice-cold radio immunoprecipitation assay (RIPA) buffer (containing a protease inhibitor and TRITON-X-100), and Homo-buffer (containing phosphatase inhibitor, vanadate (1:50)), for TRPC-1 protein and e-NOS proteins respectively. Homogenates were centrifuged (10–15 min, 12,000 rpm, 4 °C). Both proteins were resolved on an 8% sodium dodecyl sulfate (SDS)-polyacrylamide gel electrophoresis (PAGE, 1 mm gel cassette), and transferred into nitrocellulose membranes. Blots were probed overnight (4 °C, and 1% milk), with anti-TRPC-1 rabbit primary antibody (1:500, (ab192031) Abcam) or anti-e-NOS mouse primary antibody (1:250, (ab 76198) Abcam). Membranes were then incubated for 1 h at room temperature (RT) with secondary anti-rabbit antibody (1:1000, (sc-2370) Santa Cruz, TX, USA) and secondary anti-mouse antibody (1:5000, (A9044) Santa Cruz), for TRPC-1 and e-NOS respectively. Both secondary antibodies were conjugated with horseradish peroxidase (HRP) enzyme. Signals were developed by using an enhanced chemiluminescent substrate for detection of HRP (ECL Western Blotting Substrate, Thermo Scientific, Rockford, IL, USA) and exposed to Hyperfilm. Films and protein bands densities were analysed using the Image Quant Software (Amersham Pharmacia Biotech, Buckinghamshire, UK) after scanning with Gel Analyst 3.01 Software (Iconix, Toronto, ON, Canada). Blots are shown in Figure 15.

### 4.9. Protein Determination

Aliquots (20 μL) from diluted samples (15- or 25-fold with distilled water) were mixed with 980 μL of distilled water, after which 200 μL of Bradford reagent was added to each sample. After mixing and 10 min of incubation, samples were assayed spectrophotometrically at *λ* = 595 nm with a commercial protein assay kit (Bio-Rad Labs, Budapest, Hungary). Protein levels were expressed as (mg protein/mL).

### 4.10. Statistical Analysis

All data are shown as mean ± SEM. Statistical comparisons were performed with Student’s two-tailed unpaired *t* test and a multiple comparison test (Bonferroni) when necessary. Differences were considered significant when *p*-values were less than 0.05 (*p* < 0.05).

## 5. Conclusions

The findings of our study showed that pre-treatment with oral administration of a 50 mg dose of sitagliptin could reduce the myocardial injury in an I/R *ex vivo* model, by decreasing infarct size, up-regulating GLP-1 level preceded by DPP-4 inhibition thereby activating the TRP/CGRP signaling pathway, and increasing NOS activity and e-NOS expression. However, taking into account the results of the ineffective higher doses of sitagliptin that we observed in e-NOS and TRPC-1 protein expression, this issue requires further investigations in the near future, and this drug can be considered for the treatment of ischemic diseases at a higher level, only after clarifying the molecular mechanisms underlying these doses.

## Abbreviations

MI	Myocardial infarction
IR	Ischemia-reperfusion
IS	Infarct size
DPP-4	Dipeptidyl peptidase- 4
DPP-4i	Dipeptidyl peptidase-4 inhibitor
GLP-1	Glucagon-like peptide-1
NOS	Nitric oxide synthase
e-NOS	Endothelial nitric oxide synthase
i-NOS	Inducible nitric oxide synthase
n-NOS	Neuronal nitric oxide synthase
TRP	Transient receptor potential
TRPC	Transient receptor potential canonical
TRPV	Transient receptor potential vanilloid
CGRP	Calcitonin gene-related peptide
l-NAME	Nω-nitro-l-arginine methyl ester
CAP	Capsazepine
TTC	Triphenyltetrazolium chloride
LAD	Left anterior descending
AAR	Area at risk
Sitg	Sitagliptin
PBS	Phosphate buffer saline
DMSO	Dimethyl sulfoxide
